# RC48-ADC combined with tislelizumab as neoadjuvant treatment in patients with HER2-positive locally advanced muscle-invasive urothelial bladder cancer: a multi-center phase Ib/II study (HOPE-03)

**DOI:** 10.3389/fonc.2023.1233196

**Published:** 2024-01-10

**Authors:** Feng Wen, Tianhai Lin, Peng Zhang, Yali Shen

**Affiliations:** ^1^ Abdominal Oncology Ward, Division of Radiation Oncology, Cancer Center, West China Hospital, Sichuan University, Chengdu, China; ^2^ Department of Urology, Institute of Urology, West China Hospital, Sichuan University, Chengdu, China

**Keywords:** neoadjuvant treatment, HER2, muscle-invasive urothelial bladder cancer, RC48-ADC, tislelizumab

## Abstract

**Background:**

Bladder cancer with high expression of human epidermal growth factor receptor-2 (HER2) is related to pathological malignancy and poor prognosis. The standard care for muscle-invasive urothelial bladder cancer (MIBC) is neoadjuvant chemotherapy (NAC) followed by radical cystectomy (RC) with pelvic lymph node dissection. For HER2-positive MIBC, the efficacy of cisplatin-based NAC is unsatisfactory, and adverse reactions are inevitable or even intolerable. New regimens with higher efficiency and lower toxicity need to be explored in the neoadjuvant setting for this population.

**Methods:**

HOPE-03 is a multi-center, open-label, single-arm, phase Ib/II study aiming to evaluate the safety and efficacy of RC48-ADC (disitamab vedotin (DV)), a humanized anti-HER2 antibody conjugated with monomethyl auristatin E, and tislelizumab (PD-1 antibody) as a novel neoadjuvant treatment combination in patients with HER2-positive locally advanced urothelial MIBC. Fifty-one patients with cT2-4bN0-3M0-1a pathology- and imaging-diagnosed HER2 positive (immunohistochemistry status 3+ or 2+ or 1+) MIBC were recruited. Of these patients, six were enrolled in the dose-escalation phase (three patients in the RC48-ADC 1.5 mg/kg group and three patients in the 2.0 mg/kg group), and 45 patients were enrolled in the phase II study (the expected recommended phase II dose for RC48-ADC was 2.0 mg/kg). Patients without disease progression received radical cystectomy or bladder-sparing therapies as their preference after neoadjuvant treatment. The primary endpoints were clinical complete remission rate (cCR rate; T0/Ta/Tis), pathological complete remission rate (pCR rate), and safety. The secondary endpoints were overall survival (OS), local recurrence-free survival (LRFS), distant metastasis-free survival (DMFS), and quality of life.

**Discussion:**

The HOPE-03 trial provides a description of the safety profile of RC-48 and tislelizumab combination in the neoadjuvant treatment of HER2-positive locally advanced urothelial MIBC, and the efficacy is explored as well in this population.

**Clinical trial registration:**

https://www.chictr.org.cn/showproj.html?proj=137111, identifier ChiCTR2200060153.

## Introduction

Bladder cancer is the tenth most commonly diagnosed malignancy worldwide, with an estimated 573,278 new cases and 212,536 deaths in 2020 (1). It is more common in men than in women. For men, the estimated incidence and mortality rank fourth and eighth among all cancer types in American in 2022, respectively (2). Urothelial carcinoma accounts for approximately 90% of bladder cancer, and more than 30% of bladder cancers are muscle-invasive urothelial bladder cancer (MIBC) (3). Platinum-containing neoadjuvant chemotherapy (NAC) followed by radical cystectomy and pelvic lymph node dissection is a widely used standard treatment for urothelial MIBC, with a 5-year overall survival (OS) rate of approximately 50%–70% (4).

Notwithstanding the established position of NAC in urothelial MIBC, there are many challenges in the implementation process. Meta-analyses have shown there is a 5% absolute improvement in the OS at 5 years for NAC combined with local treatment compared with local treatment alone (5). In clinical settings, the data are not satisfactory enough, and NAC is dependent on some patients’ surgical tolerance. Hence, compliance with clinical guidelines in daily practice is as low as 20%. Additionally, approximately 50% of patients are not eligible for cisplatin or neoadjuvant treatment because of risk comorbidities, such as impaired renal function, heart failure, and hearing loss (4, 6). Moreover, the situation is different when it comes to human epidermal growth factor receptor-2 (HER2)-positive urothelial MIBC patients, which opens the path for precision medicine.

HER2, as a member of the epidermal growth factor receptor family, plays an important role in cell proliferation and differentiation through tyrosine kinase activity (7). As is known, the amplification of the HER2 gene results in the overexpression of HER2 protein, which is related to the growth of several tumors, such as breast cancer, gastrointestinal cancer, and ovarian cancer. Therefore, HER2 is a promising target in clinical cancer treatment. HER2 overexpression in urothelial bladder cancer varies from 4% to 76%, which is one of the most prevalent carcinomas (8–11). It is verified that the overexpression of HER2 protein is related to tumor progression and poor prognosis in bladder cancer, and the efficacy of cisplatin-based NAC in HER2-positive urothelial bladder cancer is unsatisfactory (12–14). However, the exploration of the antitumor efficacy of HER2 antibodies and tyrosine kinase inhibitors (TKIs) in HER2-positive urothelial carcinoma (UC) has encountered dilemmas (15, 16). Hence, new regimens with better efficiency and lower toxicity need to be explored in the neoadjuvant setting for this population.

RC48-ADC (disitamab vedotin (DV)), a humanized HER2 targeting antibody conjugated with monomethyl auristatin E (MMAE), is a breakthrough treatment for metastatic UC that was granted approval by the Food and Drug Administration (FDA) in 2020 based on the results of RC48-C005 and RC48-C009 studies, in which RC48-ADC demonstrated a promising efficacy with a manageable safety profile in HER2-positive locally advanced or metastatic UC (mUC) patients with tumor progression after platinum-based chemotherapy (3).

Meanwhile, immune checkpoint inhibitors (ICIs) targeting programmed cell death protein 1 (PD-1) and programmed cell death-ligand 1 (PD-L1) have significantly changed the treatment landscapes of locally advanced/unresectable or metastatic UC (17). Previous studies showed that the combination of RC48-ADC and PD-1 antibody may have synergistic antitumor effects because ADC linking to MMAE elicits immunogenic cell death (ICD) and has a direct effect on dendritic cell (DC) maturation and activation, which may enhance antitumor immunity (18, 19). Moreover, the combination of RC-48-ADC and PD-1 antibody (toripalimab) in a phase Ib/II trial (RC48-C014) confirmed a promising objective response rate (ORR) of 73.9% for metastatic UC patients without previous system treatment and a good tolerance (20).

Until now, the application of RC-48-ADC and PD-1 antibody combination in UC is limited, and most of the safety and efficacy data are from phase II trials. The combination effect may be varied with different ICIs. Moreover, the safety and efficacy of this combination as the neoadjuvant treatment for HER2-positive urothelial bladder cancer are still unknown. Therefore, a multi-center, open-label, single-arm, phase Ib/II study was designed, which is the first study to explore the safety and efficacy of RC48-ADC and tislelizumab (PD-1 antibody) as a novel neoadjuvant treatment combination in patients with HER2-positive locally advanced urothelial MIBC. Whether this setting can achieve better efficacy and safety profile compared with platinum-based NAC in the neoadjuvant treatment of HER2-positive MIBC patients will be further explored in phase III randomized controlled clinical studies after the preliminary results.

## Materials and methods

### Study design

**Figure 1:**
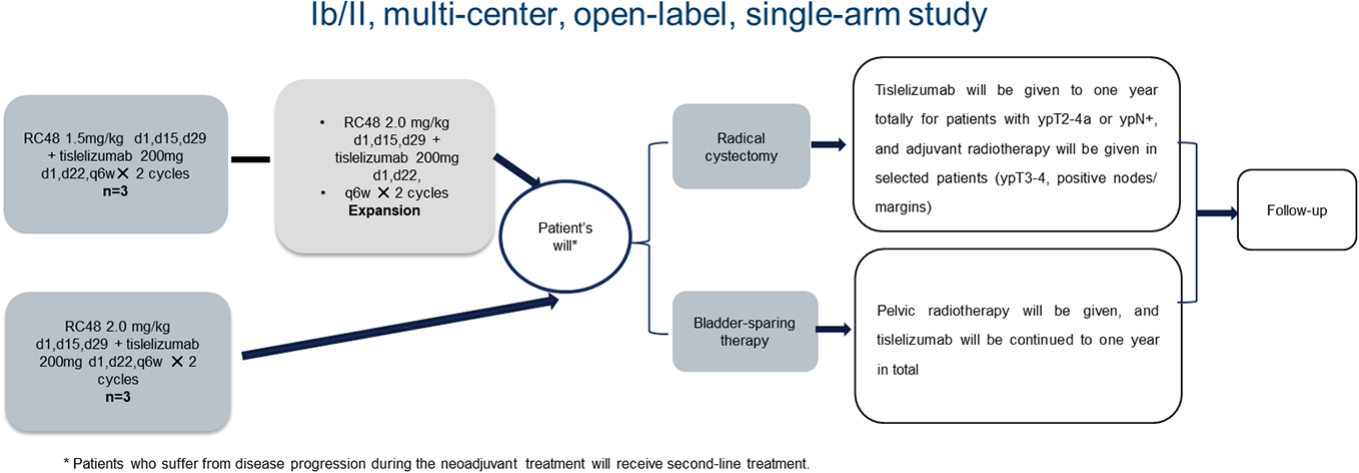
The flowchart of the HOPE-03 study.

This was a multi-center, open-label, single-arm, phase Ib/II study. Patients with cT2-4bN0-3M0-1a pathology- and imaging-diagnosed HER2-positive (immunohistochemistry status 3+ or 2+ or 1+) MIBC were recruited. Of these patients, six were enrolled in the dose-escalation phase (three patients in the RC48-ADC 1.5 mg/kg group and three patients in the 2.0 mg/kg group), and 45 patients were enrolled in the phase II study (the expected recommended phase II dose for RC48-ADC was 2.0 mg/kg). RC48-ADC was given every 2 weeks with a maximum dose of 120 mg in 60 minutes of intravenous infusion. Meanwhile, tislelizumab was given every 3 weeks at a dose of 200 mg in no less than 30 minutes of intravenous infusion. No premedication was required. Treatment efficacy was performed by imaging and transurethral multi-point biopsy after the neoadjuvant therapy. Patients without disease progression received radical cystectomy or bladder-sparing therapies as their preference thereafter. For patients receiving radical cystectomy, tislelizumab was given for a total of 1 year for patients with ypT2-4a or ypN+, and adjuvant radiotherapy was considered in selected patients (ypT3-4, positive nodes/margins). For patients who preferred bladder-sparing therapies, pelvic radiotherapy was given, and tislelizumab was continued for a total of 1 year. Patients who suffered from disease progression during the neoadjuvant treatment received second-line treatment. The flowchart of the study is presented in **Figure 1**.

### Study organization, ethics approval, and drug supply

The current trial was mainly initiated by West China Hospital, Sichuan University, and three other institutions participated in the study, including Sichuan Provincial People’s Hospital, the Affiliated Hospital of Southwest Medical University, and the Affiliated Hospital of North Sichuan Medical College. The treatment protocol was approved by the medical ethics committee of West China Hospital, Sichuan University, and the Chinese Ethics Committee of Registering Clinical Trials (ChiECRCT20210564). Signed informed consent was obtained before eligible patients were enrolled. Additionally, the study was performed in accordance with the ethical standards put forth in the 1964 Declaration of Helsinki. RC48-ADC and tislelizumab were provided free of charge by RemeGen Ltd. and BeiGene Ltd. for clinical trial subjects, respectively.

### Study population

The patients with HER2-positive urothelial MIBC were selected according to the inclusion criteria and exclusion criteria (**Table 1**). Patients who were suitable or not suitable for platinum-based NAC were included in the study. Patients who were fit or had HER2 status of 1+, 2+, and 3+ as confirmed by immunohistochemistry (IHC) and/or fluorescence *in situ* hybridization (FISH) were included in this study. A standardized HER2 scoring system for urothelial carcinoma has not been developed yet. The Tumor Pathology Committee of the Chinese Anti-Cancer Association and Expert Committee on Urothelial Carcinoma of the Chinese Society of Clinical Oncology published “Clinical pathological expert consensus on HER-2 testing in urothelial carcinoma in China” in 2021, and the scoring system was slightly modified on the basis of breast cancer HER2 detection guidelines (21). Therefore, the HER2 evaluation in the current study was based on this consensus. The status of HER2 and PD-L1 expression were stratified as analysis factors. The study was started on December 24, 2021.

**Table 1:** Inclusion and exclusion criteria.

Inclusion criteria	Exclusion criteria
1. Pathologically confirmed urothelial cancer 2. Imaging-diagnosed cT2-4bN0-3M0-1a muscle-invasive urothelial bladder cancer 3. Immunohistochemistry status of HER2 was 3+ or 2+ or 1+ 4. No prior systemic chemotherapy 5. Age ≥ 18 years, male and female 6. Eastern Cooperative Oncology Group (ECOG) physical status 0 or 1 7. Sufficient functions of heart, bone marrow, liver, kidney, and other organs 8. Good compliance and provided signed consent	1. Pregnant women or breastfeeding 2. Uncontrolled infection disease that needs systemic therapy 3. Diagnosed with other malignancies within 5 years 4. History of previous antitumor treatment, including chemotherapy, immune checkpoint inhibitor regimes, and radiotherapy 5. Active autoimmune disease or immunodeficiencies, organ transplantation history, or systematic use of immunosuppressive drugs 6. Current severe cardiac disease, renal and/or liver dysfunction 7. Severe neurological or mental illness 8. History of acute cardiac infarction or cerebral ischemic stroke occurred within 6 months 9. Human immunodeficiency virus (HIV) infection (i.e., HIV 1 to 2 antibody positive), active syphilis infection, active pulmonary tuberculosis infection 10. Active hepatitis B virus (HBV) or hepatitis C virus (HCV) infection 11. Allergic to any component of the regimens 12. Insufficient patient compliance

### Study endpoints and assessment

The primary endpoints were clinical complete remission rate (cCR rate; T0/Ta/Tis), pathological complete remission rate (pCR rate), and safety. The secondary endpoints were OS, local recurrence-free survival (LRFS), distant metastasis-free survival (DMFS), and quality of life.

Imaging evaluation was performed based on the Response Evaluation Criteria in Solid Tumors (RECIST v.1.1) and immune Response Evaluation Criteria in Solid Tumors (iRECIST). For patients with radical cystectomy, pCR was used as the main endpoint, namely, no evidence of tumor cells in resected specimens. For patients with bladder-sparing treatment, cCR was applied as the primary endpoint, defined as undetectable tumor existence after treatment by chest and abdominal computed tomography (CT), bladder magnetic resonance (MR), cystoscopy of multi-point biopsy, and even positron emission tomography/computed tomography (PET/CT) if previous imaging results were unclear, which includes the clinical efficacy of T0/Ta/Tis. CT and MR were performed using contrast. CT of the abdomen and pelvis with contrast with excretory imaging was preferred. MRI included T2, diffusion-weighted imaging (DWI), and dynamic contrast-enhanced (DCE) sequences. The imaging evaluation was assessed by a radiologist in a masked manner and investigator independently.

Adverse effects were evaluated every cycle of treatment and recorded according to the Common Terminology Criteria for Adverse Events (CTCAE) 4.0. OS was defined as the time from enrollment to death. For patients who achieved pCR or cCR, LRFS and DMFS were recorded, defined as the time from the evidence of no existence of tumor to local recurrence or distant metastasis of disease per RECIST 1.1 or death, respectively. Patients’ quality of life was evaluated based on FACT-G scales at the end of the neoadjuvant therapy and during every follow-up visit.

### Sample size

The main research purpose of this trial was to investigate the cCR rate, and it was proposed to adopt a non-randomized controlled observation study. With reference to the data in published clinical studies or the previous treatment data of our institution, the cCR rate of HER2-positive urothelial MIBC patients who received gemcitabine and cisplatin combination with or without immunotherapy as a new adjuvant treatment was approximately 23%. We expected that the cCR rate of this research scheme would be increased to 40%, and β values of 0.2 and α values of 0.05 were applied in single-stage phase Ib/II clinical trials and considered successful. The estimated lost interview rate was 10%. As a result, 51 patients were enrolled, including six patients in the phase Ib stage: three patients in the RC48-ADC 1.5 mg/kg combined tislelizumab group and three patients in the RC48-ADC 2.0 mg/kg combined tislelizumab group. A total of 45 patients were enrolled in the phase II study. The planned enrollment time was 1–2 years. Considering the interference of other factors, we included as many patients who met the inclusion criteria as possible.

### Follow-up

Based on the study protocol, patients received regular examinations at the baseline and end of the whole neoadjuvant therapy and every visit during the follow-up. Laboratory examinations were required before each cycle of neoadjuvant treatment. Regular examinations consisted of chest and abdominal CT, bladder MR, cystoscopy of multi-point biopsy, blood cell counts, liver and kidney function test, urine routine test, and FISH test. For patients who completed cystectomy or radiotherapy, regular visits were conducted every 3 months in the first 2 years and every 6 months in the next 3 years. The total duration of the study was 5 years. All the results were explained by the investigators according to their clinical practice from the perspective of patients.

## Discussion

Bladder cancer, one of the most common malignancies, has a high global societal burden. Approximately half of locally advanced MIBC patients will ultimately develop distant metastasis of disease after radical cystectomy and pelvic lymph node dissection because of the micro-metastases in the blood (22, 23). Hence, NAC followed by local therapy plays a key role in reducing recurrence rate and metastatic rate, as well as prolonging the overall survival (24). Gemcitabine and cisplatin (GC) and dose-dense methotrexate, vinblastine, doxorubicin, and cisplatin (dd-MVAC) have been widely used in clinical practice as systematic treatment of locally advanced MIBC, with a pCR rate varying from 36% to 42% (25).

In the past 10 years, the development of sequencing technologies has deepened the understanding of the pathogenesis of bladder cancer and widened the range of potential treatment options (4). On the basis of the high mutation burden of bladder cancer genomic characterization, ICIs have been recommended in the first-line and second-line treatments of selected advanced urothelial cancer patients. Moreover, the expanded applications in neoadjuvant, adjuvant, and bladder-sparing treatments have been evaluated in advanced bladder cancer patients, in which durable responses in a subset of patients have been achieved. The pCR rate for single-agent ICI or two ICI combinations in the neoadjuvant setting varied from 7% to 46%, including 42% of pembrolizumab in the PURE-01 trial, 27% of atezolizumab in the ABACUS trial, and 43% of durvalumab plus tremelimumab in the first reported dual ICI setting (26–28). Additionally, the combination of chemotherapy and ICIs as an optional neoadjuvant also has been explored with pCR rates varying from 7% to 46%.

Additionally, with the discovery of commonly expressed molecular targets in bladder cancer, targeted therapies have been developed, such as antibody–drug conjugates of HER2 and fibroblast growth factor receptor inhibitors. Several studies showed that the overexpression of HER2 was associated with tumor progression and poor prognosis (8). Additionally, compared with HER2-negative MIBC patients, HER2 overexpression patients showed therapeutic resistance to regular chemoradiation treatment (14). Based on the real-world study of chemotherapy plus immunotherapy versus chemotherapy alone as neoadjuvant treatment-guided bladder-sparing therapy for localized MIBC of our group, we found that the cCR rate was approximately 23% for HER2-positive urothelial MIBC patients who received GC chemotherapy with or without immunotherapy as a new adjuvant treatment, and related adverse reactions were inevitable or even intolerable (29). Therefore, new regimens with higher efficiency and lower toxicity need to be explored in the neoadjuvant setting for this population.

Despite the unsuccessful clinical explorations of antibodies and TKIs of HER2 in urothelial cancer, the advent of ADCs changes the treatment landscape of advanced bladder cancer. ADCs consist of a monoclonal antibody, a protease-cleavable linker, and a chemotherapeutic drug. After the interaction of the antibody and antibody target expressed on the surface of the tumor cells, the cytotoxic agent will be released inside the cells after the internalization of ADCs and cleavage of lysosomal linker, which leads to the delivery of a high dose of the chemotherapy agent. Until now, several proteins have been investigated as the ADCs’ targets, including SLITRK6, Nectin-4, Trop-2, and Her-2 (30–32).

RC-48-ADC, with a highly selective HER2 affinity, elicited promising antitumor effects in HER2-positive advanced or metastatic urothelial cancer patients with disease progression of at least one line treatment (3). In addition, the combination of RC-48-ADC and PD-1 antibody (toripalimab) achieved a satisfactory ORR of 73.9% in metastatic UC patients without previous system treatment. It is worth verifying the disease control ability of RC48-ADC and ICI combination in the neoadjuvant treatment of locally advanced MIBC patients.

The current HOPE-03 trial is a multi-center, open-label, phase Ib/II study designed to figure out the safety and efficacy of RC48-ADC and tislelizumab combination as neoadjuvant treatment in patients with HER2-positive locally advanced urothelial MIBC. Pathology- and imaging-diagnosed (immunohistochemistry status 3+ or 2+ or 1+) cT2-4bN0-3M0-1a MIBC patients were recruited, and the sample size was 52. The enrollment is currently ongoing, and six patients have been included until now. The median age is 62 years with clinical stages of T3-4aN0-3M0. Five patients were HER2(2+), and one patient was HER2(1+). Among them, four patients received a primary efficacy evaluation. Three patients achieved T0, and one patient was Tis. Hence, the cCR rate was 100%. Only one patient suffered from immune-related grade 2 myositis and grade 1 elevated transaminase adverse event, which led to treatment interruption. Additionally, RC48-ADC-related toxicities were found in three patients, including grade 1 elevated transaminase, grade 1 erythema, and grade 1 paresthesia. Until now, no treatment-related dose reduction has occurred. In terms of toxicity, the RC48-ADC combination seems safer than other ADCs, which gives us great confidence.

To conclude, neoadjuvant RC48-ADC combined with tislelizumab in patients with HER2-positive locally advanced urothelial MIBC initially showed manageable toxicities and satisfactory efficacy in the preliminary result, which will be verified by the final analysis of this multi-center, phase Ib/II study. With the implementation of HOPE-03, we want to find an optimal neoadjuvant treatment for HER2-positive patients. Because of the limited sample size, phase III, clinical, randomized control trials are needed to further validate the efficacy of the combination therapy.

## Data availability statement

The original contributions presented in the study are included in the article/supplementary material. Further inquiries can be directed to the corresponding authors.

## Ethics statement

The studies involving humans were approved by the medical ethics committee of West China Hospital, Sichuan University and the Chinese Ethics Committee of Registering Clinical Trials. The studies were conducted in accordance with the local legislation and institutional requirements. The participants provided their written informed consent to participate in this study.

## Author contributions

YS and PZ contributed the concept and design of the study. FW and TL were involved in the organization of the trial and drafting the manuscript. All authors have given final approval of the version of the protocol, and it was also approved by local investigators at the participating centers.
